# Mysterious Epidemie in Paris, Bearing Some Resemblance to the Dandy Fever

**Published:** 1829-01-01

**Authors:** 


### LA CHARITE.

#### I. MYSTERIOUS EPIDEMIC IN PARIS.

The DANDY FEVER (of which some curious particulars will be found in the present number) has scarcely excited greater interest in the West Indies, than does an unnamed, and apparently unnameable epidemic (which has recently made its debut) in Paris. It was first observed in a particular locality, the Infirmary of Maria Theresa, but has since spread to various quarters of the city. Dr. Mequel is employed in drawing up an account of this epidemic; but, in the mean time, some interesting particulars have been reported from the wards of M. Cayol, in LA CHARITÉ. Five patients affected with the disease have been received there, of which one is dead—one has recovered—and three are still confined to their beds. We shall present our readers with some sketches of these cases.

*Case* 1. This was the Infirmary-man of the MARIA THERESA above-mentioned, who entered LA CHARITÉ on the 5th of July. He is 42 years of age, and is the first person who has had the disease, as far as is yet known. He had previously enjoyed excellent health. On the 4th of June, and without any appreciable cause, he was seized with general malaise, followed by shiverings, vomiting, violent head-ache. The throat became sore, as also the eyes, while the eyelids and the whole face became swelled and œdematous. There was considerable fever and much agitation. *Venesection—leeches to the neck and temples—low diet.* During ten or twelve days, this state continued, without any amelioration, in despite of antiphlogistic treatment the most active. At this period, the symptoms subsided, and in two or three days, they were nearly gone, when, all at once, the patient was seized with acute pains, which he compared to "traits de feu," in different members, sometimes fixing themselves on the feet, sometimes on the hands, which then became the seat of the most insupportable sensations of formication and pricking—"de formication et de picottemens." These new phenomena became daily more alarming—especially in the night, when the patient's torments were terrible, and the burning heat of his feet allowed him no repose. The medical officers of the Infirmary suspecting some affection of the spinal marrow, had applied cupping-glasses along the spine, and afterwards blisters, without any relief. He was then carried to LA CHARITÉ. The poor fellow was, by this time, completely deprived of the use of his limbs, while the sensibility of some of his members was so excessively and morbidly acute, that the slightest touch threw him into convulsions. The pains indeed were not so acute as at the beginning; but they came on in paroxysms with very short intervals. The annoying sense of formication and pricking still continued unabated in the hands and feet. There was now no fever, no head-ach—the appetite was very keen.

M. Cayol, (who had been physician to the Infirmary where the patient was first taken ill) still continued to think that the spinal marrow was affected, and ordered moxas to the neighbourhood, but without the least success. The periodicity of the paroxysms next attracting his attention, he prescribed the sulphate of quinine in very large doses, but still without producing the least relief. Opium was then tried, the dose being gradually increased to seven or eight grains a day. No good effects resulted—and on the 23d of August, when the report closed, the patient was in nearly the same state as when he entered the hospital on the 5th July!

*Case* 2. This was the case which terminated fatally. He had been ill for three months. The patient was a man about 40 years of age, a coal-heaver, inhabiting the "PLACE DE GREVE," a damp and unhealthy situation on the banks of the Seine. His constitution had been much deteriorated by the misery of his life, when he was seized, like the former patient, with shiverings, vomiting, fever, œdematous swelling of the face, &c. His wife and daughter became affected in the same way, and about the same time. They all attributed their illness to the dampness and wretchedness of their situation, and continued their avocations for eight or ten days; when acute pains in their limbs—formication and prickings—burning heat in the hands and feet—excessive debility, &c. confined them to their beds, where they lay upwards of two months, before they determined on claiming the resources of an hospital. The man was carried to La Charité, and, although he did not then present any of the phenomena of fever, yet his general aspect indicated a fatal result. His lower extremities were wasted, and incapable of voluntary motion—their surface covered with furfuracious scales, and of a dark earthy colour—hands in the same state—face no longer œdematous; but his eyes were lachrymose and very painful. A few days after this, the patient's daughter was able to pay her father a visit, and, on this occasion, he became greatly agitated. The next day, fever was kindled up, accompanied by harrassing cough, and copious expectoration. The chest sounded well, and the respiration was heard, without "râle;" but there was deep-seated pain in the thorax, and two small bleedings were employed. The blood was not inflamed; and the thoracic affection was increased rather than diminished. The tartrite of antimony was then employed in large doses—eighteen grains in the 24 hours. There was a momentary mitigation of the pulmonary affection; but the fever increased, and the patient soon died delirious.

*Dissection.* The lower portions of the lungs were gorged with a black and fetid serosity; but otherwise crepitous, and permeable by the air. Heart flaccid and soft. The liver presented a remarkable appearance, being, as it were, crisped up, or shrivelled. The spleen was enlarged to double its natural size. The most accurate examination did not detect any organic lesion in any of the viscera of the head, spine, or abdomen.

*Case* 3. This was the patient cured. The symptoms were the same in kind, but slighter in degree. His wife was affected with the same complaint, but they both recovered.

*Cases* 4 *and* 5. These are both females. They have been ill more than two months, with this strange complaint. The second of these females presents phenomena which are more remarkable than in the other. The patient is 29 years of age, and entered the hospital on the 14th of August, being then six weeks ill of the complaint, which commenced in the way already described. To the usual symptoms was added a most obstinate and *constant* cough, without any pain in the chest. In the course of a few days, this catarrh took on a new character. The cough became *periodical,* occurring in violent paroxysms, several times in the day, and was then indescribedly distressing. The patient felt as if all the bones of the thorax were breaking; while the extremities were contracted, convulsed, and the seat of a burning pain. These paroxysms were always preceded by a chill or rigor, which sometimes lasted three quarters of an hour, terminating in vomiting. In the intervals, the patient felt overwhelmed with weakness, and complained of a sense of formication and pricking in the hands and feet. These symptoms continued for about three weeks, and then became gradually mitigated. The skin is quite black on the chest and abdomen—the face swelled and œdematous—the eyes lachrymose. Latterly the patient has been free from the paroxysms of cough; but every morning she had a cold chill, which ushered in some febrile movement of an aguish character. Before she entered La Charité, she took no medicines. Since then, she had an emetic, and afterwards opium and quinine. After taking the bark, for a short time, sleep returned, the febrile accessions are scarcely perceptible—the cough is gone—and, in fact, she is nearly well.

The foregoing epidemic, we think, will puzzle our systematic nosologists! We know of no other probable etiology than that which we are certain Dr. Macculloch would give—a malaria. The intermittent character which it has decidedly assumed in some of the cases at least—and the neuralgic character manifested in all of them, render it highly probable that the disease is of malarious origin—and of the neuralgic order.

